# A Case of Levamisole-Induced Agranulocytosis

**DOI:** 10.1155/2018/7341835

**Published:** 2018-02-11

**Authors:** Thamer Kassim, Lakshmi Chintalacheruvu, Osman Bhatty, Mohammad Selim, Osama Diab, Ali Nayfeh, Jayadev Manikkam Umakanthan, Maryam Gbadamosi-Akindele

**Affiliations:** ^1^Department of Internal Medicine, Creighton University, Omaha, NE, USA; ^2^University of Nebraska Medical Center, Omaha, NE, USA

## Abstract

A sixty-eight-year-old male with a past medical history of recurrent cocaine use presented to the emergency department with recurrent diarrhea and was found to have a white blood cell (WBC) count of 1.9 × 10^9^/L with agranulocytosis (absolute neutrophil count (ANC) of 95 cell/mm^3^). At admission, the patient disclosed that he used cocaine earlier during the day, and a urine drug screen tested positive for this. On hospital day one, the patient was found to have a fever with a maximum temperature of 313.6 K. After ruling out other causes and noting the quick turnaround of his neutropenia after four days of cocaine abstinence, the patient's neutropenia was attributed to levamisole-adulterated cocaine.

## 1. Introduction

Levamisole is an immunomodulator that is found in almost seventy to eighty percent of cocaine shipments according to the Drug Enforcement Agency (DEA) [1, 2]. There were approximately 1.5 million American cocaine users each month in the year of 2013 making levamisole a relevant public health concern [3]. We present a case of levamisole-cocaine-induced agranulocytosis with the aim to raise awareness regarding the possible complications of this compound.

## 2. Case Report

A sixty-eight-year-old male presented to the Emergency Department (ED) complaining of recurrent diarrhea and a documented fever of 312 K for one-day duration. The patient had a past medical history of end-stage renal disease on hemodialysis, insulin-dependent type II diabetes mellitus, essential hypertension, and chronic cocaine dependence. On further history, the patient reported the use of cocaine over the past five years with an average use of three times a week but could not specify a certain amount on each time. The patient disclosed that he had used cocaine earlier that day.

At admission, blood pressure was 106/78, heart rate was 96 beats per minute, respiratory rate was 18, temperature was 312.2 K, and oxygen saturation was 93% on room air. Physical examination showed a thin, malnourished male with right below knee amputation who was in mild distress. No cutaneous manifestations were noticed, and no other abnormalities were appreciated on the rest of the patient's physical examination. Laboratory workup was done and included a complete blood count (CBC) with differential, which showed a WBC count of 1.9 × 10^9^/L (reference range: 4.0–11.0 × 10^9^/L), segmented neutrophils 4% (reference range: 40–70%), bands 1% (reference range: 0–6%), immature granulocytes of 0.0% (reference range: 0.0–0.9%), and lymphocytes 70% (reference range: 16–45%). Basophils, eosinophils, and monocytes were within normal limits. Hemoglobin was 9.8 g/dL (reference range: 12.5–17 g/dL), which was around baseline and was attributed to the patient's end-stage renal disease, and platelet count was 237 × 10^9^/L (reference range: 150–450 × 10^9^/L). A complete metabolic profile was also obtained showing a serum potassium of 3.1 mmol/L (reference range: 3.6–5.0 mmol/L), creatinine of 371.4 *µ*mol/L (reference range: 45–90 *µ*mol/L), blood urea nitrogen of 9.64 mmol/L (reference range: 2.5–7.1 mmol/L), and glomerular filtration rate of 12 with a normal liver function. Urine drug screen was positive for cocaine. Erythrocyte sedimentation rate, C-reactive protein, and urine analysis were within reference range. At that point, isoantibodies including cytoplasmic antineutrophil cytoplasmic antibody (C-ANCA) and perinuclear antineutrophil cytoplasmic antibody (P-ANCA) were not tested because the patient had normal inflammatory markers and no urinary, pulmonary, or cutaneous manifestations.

The patient was admitted to the hospital, and intravenous fluids were initiated along with correction of electrolyte disturbances and symptomatic therapy. The patient's diarrhea resolved during his first admission day, but the patient spiked a fever with a temperature maximum of 313.6 K. A repeat CBC with differential was done and showed a WBC count of 1.0 × 10^9^/L, segmented neutrophils of 3%, bands of 1%, immature granulocytes of 0.0%, and lymphocytes of 44% with normal basophils, eosinophils, and monocytes. The patient's calculated ANC was 40 cells/mm^3^, and the decision to start empirical antibiotics and initiate a septic workup was taken. Blood cultures, urine analysis and culture, and stool analysis and culture were all negative. Chest X-ray and chest/abdomen CT scan showed no abnormalities, and ultimately no source of infection was isolated.

**Figure 1:**
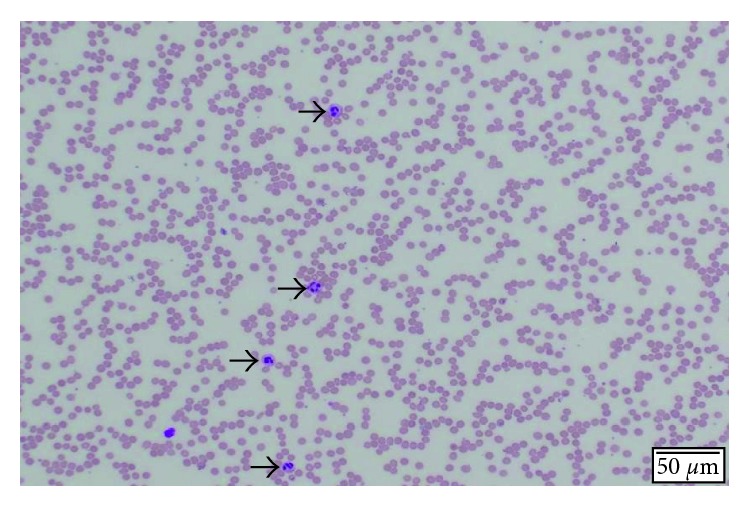
Unremarkable neutrophils in the background of anemia. Absolute leukopenia with absolute neutropenia. Black arrows show a decreased number morphologically.

Further tests were initiated in an attempt to find a cause for the patient's unexplained neutropenia. Medications were reviewed carefully for possible idiosyncratic drug reactions and bone marrow toxicity, but none of the patient's medications fit such a profile. A viral panel including hepatitis A, B, and C and HIV were all negative. Vitamin B12 level was 681.7 pmol/L (reference range: 216–687 pmol/), folate level was 38.7 nmol/L (reference range: 4.5–45.3 nmol/L), copper level of 11.2 *µ*moI/L (reference range: 9.9–23.1 *µ*moI/L), reticulocyte count of 2.3% (reference range: 0.5–2.3%), lactate dehydrogenase level of 380 IU/L (reference range: 313–680 IU/L), and negative antinuclear antibody test. A peripheral smear showed a decreased number of morphologically unremarkable neutrophils in the background of anemia (Figure 1). The patient refused a bone marrow biopsy.

**Figure 2:**
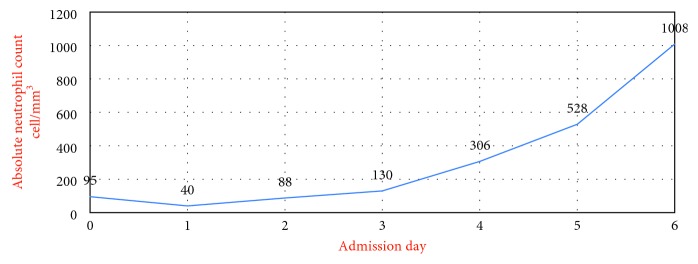


On the fourth hospital admission day, the patient started to recover on empirical antibiotics and supportive care (see Table 1 and Figure 2, resp.). The thought of an adulterant in cocaine was brought up as the patient's WBC started to improve after abstinence of drug use. Blood and urine testing for levamisole were not performed due to the short half-life of the drug and the fact that it would not have been detected at that point.

Given the patient's clinical picture, laboratory findings, and no alternative cause of his agranulocytosis, the diagnosis of levamisole-cocaine-induced neutropenia was thought to be most appropriate as a diagnosis of exclusion. The patient's white blood cell count and absolute neutrophil count started to recover four to five days after stopping cocaine use which suggested an association.

## 3. Discussion

Levamisole is a broad-spectrum antihelminthic which was first introduced in 1966 and is used currently in veterinary medicine. In humans, the drug was found to have immune-stimulant and anti-inflammatory properties and has been used for the treatment of rheumatoid arthritis, nephrotic syndrome, and other autoimmune diseases. It also showed to be beneficial in the management of lung, colon, and breast cancer. The drug was withdrawn from the US market in 1999 due to safety issues [1, 2]. For the last decade, many cases of levamisole-adulterated cocaine have been reported. These case reports suggested various complications of levamisole including neutropenia, vasculitis with cutaneous involvement, and neurological manifestations [1]. We consider the severe neutropenia in our case to be caused by cocaine adulterated with levamisole after other causes of neutropenia have been excluded.

In assessing similar patients with unexplained neutropenia, it is important to evaluate for idiosyncratic drug reactions. Reviewing new medications which can possibly cause neutropenia such as clozapine, trimethoprim/sulfamethoxazole, sulfasalazine, propylthiouracil, and methimazole can guide diagnosis [4]. When starting a workup in such cases, a differential diagnosis usually includes viral infections such as hepatitis B or C and human immunodeficiency virus (HIV) and atypical presentations of autoimmune diseases such systemic lupus erythematosus (SLE). Myelodysplastic syndrome and aplastic anemia rarely present with isolated neutropenia, but it is still important to keep an open mind for such etiologies. Other causes that need to be evaluated include deficiencies in vitamin B12, folate, and copper. Such causes were all excluded in our case.

The pathogenesis behind neutropenia caused by levamisole is not fully understood [1]. Levamisole is thought to form antigen-antibody complexes that deposit on the surface of neutrophils causing complement fixation, activation, and cytolysis. It increases T-cell activation and proliferation and increases neutrophil mobility, adherence, and chemotaxis [1]. Also, levamisole acts as a hapten increasing the formation of antibodies to granulocyte antigens and triggering an immune response resulting in destruction of leukocytes [1, 4]. Another explanation is that it could serve as a substrate for myeloperoxidase to form reactive metabolites that might stimulate autoimmunity [5].

Studies have shown that levamisole use has been associated with multiple isoantibodies including C-ANCA and P-ANCA. Antibodies to double-stranded DNA, cardiolipin, lupus anticoagulant, ribonucleoprotein, antiphospholipids, and antineutrophil cell wall antibodies have also been detected [4, 6]. The presence of such antibodies correlates with the adverse manifestations of levamisole use [1, 4]. Also, research has shown that agranulocytosis recurs with recurrent cocaine use. Genotyping reveals a significant association between HLA-B27 and cocaine-associated agranulocytosis (odds ratio (OR) 9.2; 95% confidence interval (CI)). This does not mean that every patient needs to be tested for genetic predisposition, but clinicians should have a high index of suspicion in these patients and warn them about the possible risks [7].

It is still not known why drug dealers add levamisole to cocaine. Inert adulterants are added to illicit drugs by drug dealers to increase its weight and volume before sale [1]. It has been suggested that levamisole has similar physical properties as cocaine as it can increase norepinephrine transmission through inhibition of norepinephrine reuptake and can be partially metabolized to amphetamine-like compound. Also, it can increase opioid and dopamine concentrations in the cerebral reward pathway making it a favorable adulterant to cocaine potentiating its effect [8, 9].

Levamisole levels can be detected in urine and plasma by gas chromatography mass spectroscopy. The timing is important since the half-life of levamisole is only 5.6 hours and remains detectable for 2-3 days after initial exposure [5, 7]. Bone marrow biopsy in such patients reveals a hypercellular bone marrow with a relative myeloid hypoplasia.

The most common treatment of levamisole-induced neutropenia is the withdrawal of the drug. Granulocyte colony-stimulating factor use results in neutrophil recovery within 2-3 days of administration. However, spontaneous neutrophil recovery after 5–10 days of cessation of levamisole has been previously reported [10]. Supportive care during the period of agranulocytosis and having a low threshold in initiating antimicrobials is of high importance and can prevent life-threatening opportunistic infections.

## 4. Conclusion

Levamisole should always be on the list of differential diagnoses in patients who are dependent on cocaine and are found to have neutropenic fever. Clinicians should always keep in mind early testing for levamisole in suspected cases. Such patients are at risk of life-threatening opportunistic infections, and an infectious workup together with broad-spectrum antibiotics should be initiated immediately.

Levamisole-cocaine immune-mediated disease is a public health concern in cocaine users. In light of the high number of cocaine users along with the fact that almost 70 to 80% of cocaine has been adulterated with levamisole, physicians should counsel patients about the adverse effects of such compounds. Patients should be aware of the possible signs and symptoms and should be counselled about the increased recurrence risk of these manifestations with continued cocaine use.

## Figures and Tables

**Table 1:** 

Day	1 month prior to admission	0	1	2	3	4	5	6
White blood cell count (4.0–11.0 × 10^9^/L)	4.3	1.9	1.0	1.1	1.3	1.8	2.4	3.6
Absolute neutrophil count (ANC), cell/mm^3^	—	95	40	88	130	306	528	1008
White blood cell differential (%)								
Segmented neutrophil % (43.0–76.0)	41	4	3	8	9	13	18	23
Bands % (0–6)	0	1	1	0	1	4	4	5
Lymphocyte % (16.0–45.0)	28	70.7	50	52	57.3	63	46	42
Monocyte % (5.0–14)	8	14	12	12	18	16	11	10
Eosinophil % (0.0–5.0)	0	4.0	4.8	4.5	4.2	3.3	3.0	2.5
Basophil % (0.0–1.0)	0	1.0	1.0	0.5	0.7	0.6	0	0

## References

[1] Buchanan J. A., Lavonas E. J. (2012). Agranulocytosis and other consequences due to use of illicit cocaine contaminated with levamisole. *Current Opinion in Hematology*.

[2] Bertol E., Mari F., Milia M. G., Politi L., Furlanetto S., Karch S. B. (2011). Determination of aminorex in human urine samples by GC-MS after use of levamisole. *Journal of Pharmaceutical and Biomedical Analysis*.

[3] Drugabuse.gov (2017). Nationwide trends. https://www.drugabuse.gov/publications/drugfacts/nationwide-trends.

[4] Andersohn F., Konzen C., Garbe E. (2007). Systematic review: agranulocytosis induced by nonchemotherapy drugs. *Annals of Internal Medicine*.

[5] Dherange P. A., Beatty N., Al-Khashman A. (2015). Levamisole-adulterated cocaine: a case of retiform purpura, cutaneous necrosis and neutropenia. *BMJ Case Reports*.

[6] Arora N. P., Jain T., Bhanot R., Natesan S. K. (2012). Levamisole-induced leukocytoclastic vasculitis and neutropenia in a patient with cocaine use: an extensive case with necrosis of skin, soft tissue, and cartilage. *Addiction Science & Clinical Practice*.

[7] Buxton J. A., Omura J., Kuo M. (2015). Genetic determinants of cocaine-associated agranulocytosis. *BMC Research Notes*.

[8] Larocque A., Hoffman R. S. (2012). Levamisole in cocaine: unexpected news from an old acquaintance. *Clinical Toxicology*.

[9] Brunt T. M., van den Berg J., Pennings E., Venhuis B. (2017). Adverse effects of levamisole in cocaine users: a review and risk assessment. *Archives of Toxicology*.

[10] Zhu N. Y., Legatt D. F., Turner A. R. (2009). Agranulocytosis after consumption of cocaine adulterated with levamisole. *Annals of Internal Medicine*.

